# Terminology describing the profession of Diagnostic Radiography

**DOI:** 10.1002/jmrs.361

**Published:** 2019-10-22

**Authors:** Rob Davidson

**Affiliations:** ^1^ Diagnostic Radiographer University of Canberra Bruce ACT 2617 Australia

I have recently read the article by Hooper T et al, *Dose reduction in CT imaging for facial bone trauma in adults: A narrative literature review*, published in February 2019 edition of Journal of Medical Radiation Sciences^1^. It is good to see student or recent graduate articles, in this case a narrative literature review, being published. Such work develops professional approaches and the profession itself.

I do have a significant concern. Diagnostic Radiography in both Australian and New Zealand, and in many other countries, is a health profession. In some other countries, radiographers/radiologic technologists are still trying to gain full recognition as a profession from their other medical and health colleagues. The Australian Council of Professions defines a profession as ‘possessing special knowledge and skills in a widely recognised body of learning derived from research, education and training at a high level’,^2^ and Hashimoto includes detailed discussion on professional autonomy^3^. There are many articles discussing aspects of professionalism in Diagnostic Radiography, several of which are cited here^4^, ^5^, ^6^, ^7^. Diagnostic Radiography meets the criteria of being a profession, including autonomy, and we should continually assert that we are a profession.

The article by Hooper *et al* refers to radiographers as ‘technicians’. They also imply in their conclusion that ‘technicians’ must work with radiologists on dose reduction. This statement to me lacks autonomy in our role as professionals. I have been fortunate to be able to research in and have had many discussions with radiographers around dose reduction. I have seen the professional characteristics of many radiographers including their autonomy in dose monitoring and reduction.

I commend the importance of the article for information on and approaches to dose reduction in CT imaging of facial bones. Unfortunately, the article diminishes the profession of Diagnostic Radiography with the use of such terminology.
